# dbNSFP v4: a comprehensive database of transcript-specific functional predictions and annotations for human nonsynonymous and splice-site SNVs

**DOI:** 10.1186/s13073-020-00803-9

**Published:** 2020-12-02

**Authors:** Xiaoming Liu, Chang Li, Chengcheng Mou, Yibo Dong, Yicheng Tu

**Affiliations:** 1 USF Genomics & College of Public Health, University of South Florida, Tampa, FL USA; 2 Department of Computer Science and Engineering, College of Engineering, University of South Florida, Tampa, FL USA

**Keywords:** Whole exome sequencing, Database, Nonsynonymous SNV, Deleteriousness prediction, Functional annotation

## Abstract

Whole exome sequencing has been increasingly used in human disease studies. Prioritization based on appropriate functional annotations has been used as an indispensable step to select candidate variants. Here we present the latest updates to dbNSFP (version 4.1), a database designed to facilitate this step by providing deleteriousness prediction and functional annotation for all potential nonsynonymous and splice-site SNVs (a total of 84,013,093) in the human genome. The current version compiled 36 deleteriousness prediction scores, including 12 transcript-specific scores, and other variant and gene-level functional annotations. The database is available at http://database.liulab.science/dbNSFP with a downloadable version and a web-service.

**Supplementary information:**

The online version contains supplementary material available at 10.1186/s13073-020-00803-9.

## Background

Whole-exome sequencing (WES) and whole-genome sequencing (WGS) have been increasingly used in human disease studies in the research and clinical setting [1–3]. As a result, we witness a tsunami of DNA sequence data from both healthy individuals and those with Mendelian or complex diseases. Identifying variants that cause diseases or are associated with disease risks from a large number of DNA variants identified in sequencing requires an excessive amount of time and effort. To accomplish this daunting task, investigators have relied on functional annotations to filter or prioritize variants based on our current knowledge or prediction models. In more detail, functional annotations can be separated into general annotation and functional prediction: the former provides qualitative or descriptive annotation of a variant indirectly related to its potential function, such as whether the variant is a nonsynonymous SNV; the latter typically provides direct quantitative or yes-or-no deleteriousness prediction of the variant based on a statistical model. Fast and comprehensive functional annotation tools will become even more critical in the near future because of three intertwined ongoing trends: the decreasing cost of DNA sequencing, the development and practice of precision medicine [4], and the adaptation of WES and WGS in small labs [5].

There have been several annotation tools available for large-scale DNA sequence data, such as UCSC Genome Browser’s Variant Annotation Integrator [6], Ensembl’s Variant Effect Predictor (VEP) [7], ANNOVAR [8], and SnpEff [9]. Most of these focused on general annotations based on given gene models. Although gene-model based annotations are handy, there are other important functional annotation resources used by medical geneticists and genetic epidemiologists, including functional prediction of variants, conservation information, predicted promoters, enhancers, and epigenomic markers, among others. Another challenge faced by the investigators is that different gene-model-based annotation tools all have their advantages and disadvantages, and the results sometimes do not agree with each other [10]. Therefore, it has been suggested to obtain annotation from tools across multiple databases for a complete interpretation of the variants. Previously, we developed dbNSFP version 1 [11], 2 [12], and 3 [13] to provide a “one-stop-shop” for functional annotations for non-synonymous SNVs (nsSNVs) and splice site SNVs (ssSNVs), top candidate variant types for Mendelian diseases. It collected all possible nsSNVs and ssSNVs based on human reference sequences and multiple deleteriousness predictions and annotations for each SNV.

Here we report the major updates of dbNSFP since version 3.0 to the current version 4.1. The core SNVs have been rebuilt based on human reference sequence version hg38 and GENCODE version 29 [14]. Compared to version 3.0 [13], dbNSFP v4.1 added 18 deleteriousness prediction scores (BayesDel_addAF and BayesDel_noAF [15], CADD_hg19 [16], ClinPred [17], DEOGEN2 [18], Eigen and Eigen PC [19], FATHMM-XF [20], GenoCanyon [21], LINSIGHT [22], LIST-S2 [23], M-CAP [24], MPC [25], MutPred [26], MVP [27], PrimateAI [28], REVEL [29], SIFT4G [30]), one score for loss of function prediction (ALoFT [31]), and three conservation scores (phyloP17way_primate [32], phastCons17way_primate [33], bStatistic [34]), making the total number of prediction scores to 46 (Additional file 1: Table S1). Many other functional annotation resources have been added or updated. In addition to the previously supported query of two attached databases, dbscSNV [35] and SPIDEX [36], for predicting splice interrupting SNVs, the companion query program for the downloadable version added support for querying SpliceAI, a third-party database for predicting splice site gain and loss [37], and dbMTS, a comprehensive database for microRNA target site SNVs and their functional predictions [38]. More importantly, much effort has been made to increase further the usability of the functional annotations, including (1) making functional predictions transcript-specific whenever possible, (2) providing transcript annotations to help to choose appropriate transcript from multiple isoforms for each gene, (3) providing HGVS (Human Genome Variation Society) c. and p. presentations of the SNVs to facilitate the query of candidate mutations reported in medical genetics literatures, and (4) providing graphic user interface for querying downloadable version as well as web-service for researchers with minimum bioinformatics training.

## Construction and content

We rebuilt the list of all potential nonsynonymous and splice-site SNVs based on the GENCODE gene model version 29 (Ensembl version 94) with human reference sequence GRCh38. Only transcripts with complete protein-coding annotations were included. A total of 81,782,923 nsSNVs and 2,230,170 ssSNVs were collected in the database (Additional file 2: Table S2). The corresponding chromosomal positions of the SNVs based on human reference sequences hg19 and hg18 were obtained via the liftover tool [39] (Additional file 2: Table S2). Accurate protein ID mapping between GENCODE/Ensembl and Uniprot [40] was obtained via a comprehensive protein sequence matching between all the proteins in GENCODE/Ensembl and those of the Uniprot database. To facilitate the choice of the appropriate transcript(s) for each gene, we collected transcript quality measures including APPRIS [41], transcript support level (TSL), GENCODE Basic, and Ensembl canonical transcripts were obtained from the Ensembl Biomart [42] and Variant Effect Predictor (VEP). HGVS c. and p. presentations by ANNOVAR, snpEff, and VEP for each nsSNV and ssSNV were obtained via the WGSA (WGS Annotator) pipeline [43]. As a core content of dbNSFP, 36 deleteriousness prediction scores, nine conservation scores, and one loss of function score for each nsSNV or ssSNV were compiled (see Additional file 1: Table S1 for a summary). Among them, 13 scores are transcript-specific (ALoFT, DEOGEN2, FATHMM [44], LIST-S2, MPC, MutationAssessor [45], MVP, Polyphen2 HDIV and Polyphen2 HVAR [46], PROVEAN [47], SIFT [48], SIFT4G, VEST4 [49]). The full list of annotation resources and the description of all columns in dbNSFP can be found at http://database.liulab.science/dbNSFP.

## Utility and discussion

### Query utility

dbNSFP v4.1 can be accessed as either a downloadable and standalone version, or as a web-service at http://database.liulab.science/dbNSFP. The standalone version is suitable for a large-scale query, such as quickly identifying nsSNVs and ssSNVs from exome sequencing studies. As no internet connection is required, maximum speed and security can be achieved. The query can be conducted via the companion Java program, which supports both the command-line and graphic user interface (GUI). The query term can be either a genomic position (chromosome, position), an SNV (chromosome, position, reference allele, alternative allele), an amino acid (AA) change (chromosome, position, reference allele, alternative allele, reference AA, alternative AA), a dbSNP ID (rs number), an HGVS c. or p. presentation of a mutation, or a gene name or ID. The companion Java program also supports searching attached databases along with dbNSFP, including dbscSNV, SPIDEX, spliceAI, and dbMTS, which helps to identify candidate disease-causing SNVs affecting splicing and miRNA binding.

The web-service, which is managed by Microsoft SQL Server 2017, is suitable for a small-scale query such as obtaining functional annotations for candidate SNVs. By submitting one or multiple genome coordinates (chromosome, position, reference allele, and alternate allele), users can easily retrieve all the annotation columns in dbNSFP. The output will be displayed on the web page and available as a downloadable TAB-delimited text file for further filtering.

### Comparison of prediction scores

dbNSFP is in a unique position for comparing different deleteriousness prediction scores and conservation scores across the whole exome. Among the 36 deleteriousness prediction scores, the average missing rate is 11% (Additional file 2: Table S2). MVP has the lowest missing rate (0.028%); three scores have missing rates > 20%: ClinPred (21.7%), MutationAssessor (22.2%), LINSIGHT (97.7%). The very high missing rate of LINSIGHT is due to that it was designed for noncoding variants. For the 9 conservation scores, the average missing rate is 0.6%, with minimum 0.01% (phyloP100way_vertebrate and phastCons100way_vertebrate) and maximum 1.8% (bStatistic) (Additional file 2: Table S2).

**Fig. 1:**
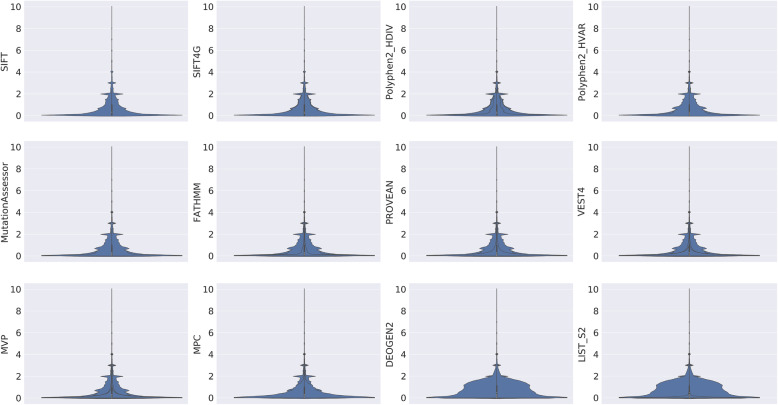
Violin plots of the dispersal statistic *d* for 12 transcript-specific deleteriousness prediction scores. *d* is capped at 10

We first compare the dispersal of the scores for the same nsSNV affecting multiple transcripts, for the 12 transcript-specific deleteriousness prediction scores. In more details, for each nsSNV affecting more than one transcript, we calculate $d=\frac{\max -\min }{\mathrm{ave}}$, where max, min, and ave are the maximum, minimum, and average of all transcript-specific scores. Of all the scores except FATHMM, there are sizable proportions of nsSNVs with a *d* > 2, suggesting that choosing an appropriate transcript is essential for predicting the impact of the SNVs (Fig. 1).

Then we compared the distribution of the scores. Because different score has a different scaling system, we create a rank score for each score so that it is comparable between scores [13]. The rank score has a scale 0 to 1 and represents the percentage of scores that are less damaging in dbNSFP, e.g., a rank score of 0.9 means the top 10% most damaging. We calculated the density distribution of the rank scores of 45 deleteriousness prediction scores and conservation scores (Additional file 3: Fig. S1, Additional file 2: Table S3). While most scores are in general evenly distributed, some scores are notably spiky and sparsely distributed, such as LRT [50], MutationTaster [51], GenoCanyon, phastCons100way_vertebrate, and phastCons30way_mammalian, among others.

**Fig. 2:**
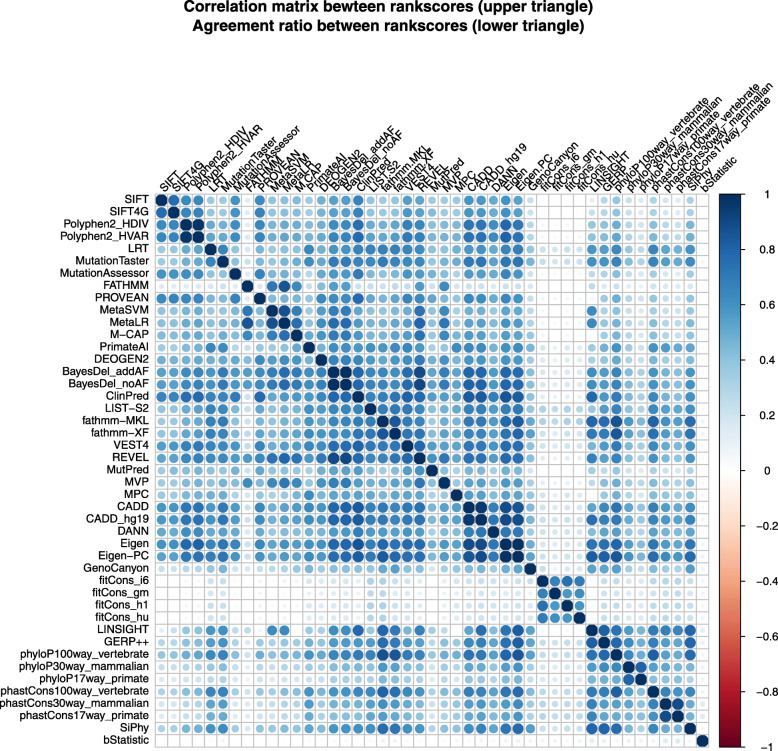
Pearson’s correlation coefficients of rank scores (upper triangle) and agreement ratio of binary predictions (lower triangle) between pairs of deleteriousness prediction scores or conservation scores

**Fig. 3:**
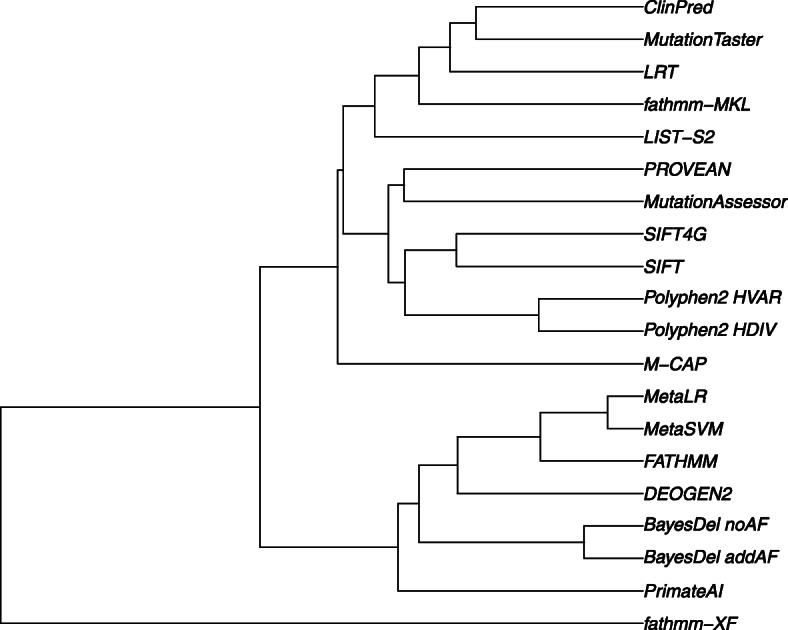
UPGMA dendrogram of the deleteriousness prediction scores and conservation scores

We also compared the correlation between scores. For the 45 deleteriousness prediction scores or conservation scores, we calculated Pearson’s correlation coefficients (*r*) of their rank scores (Fig. 2, Additional file 2: Table S4). About 43.4% of the correlations are strong (> 0.5), and 26.7% of the correlations are medium (0.3–0.5). It is noticeable that the fitCons scores have a weak correlation with other scores, except between themselves. bStatistic has weak correlations with all other scores, suggesting that the strength of background selection it measured is quite different from other conservation scores. Using 1-*r* as a distance measure, we constructed a UPGMA (Unweighted Pair Group Method with Arithmetic Mean) dendrogram of the scores (Fig. 3). Interestingly, the ensemble scores or hybrid ensemble scores in dbNSFP form two separated clusters: cluster 1 includes CADD and CADD_hg19, ClinPred, BayesDel_addAF, BayesDel_noAF, and REVEL; cluster 2 includes MetaLR and MetaSVM [52], M-CAP, and DEOGEN2. This observation suggests that they captured different features of nsSNVs or weighted the features differently.

We also compared the agreement ratio of binary predictions by 20 deleteriousness prediction scores (Fig. 2, Additional file 2: Table S5). The median agreement ratio is 0.65, which is reasonably high. Some of the highest agreement ratios are using the same training data, such as MetaLR and MetaSVM (0.96), BayesDel_addAF and BayesDel_noAF (0.94), Polyphen2_HDIV and Polyphen2_HVAR (0.88). On the other hand, some scores with similar algorithms do not have high agreement ratios: such as fathmm-XF and fathmm-MKL [53] (0.46). Fathmm-XF does not have a > 0.5 agreement ratio with any other scores.

**Fig. 4:**
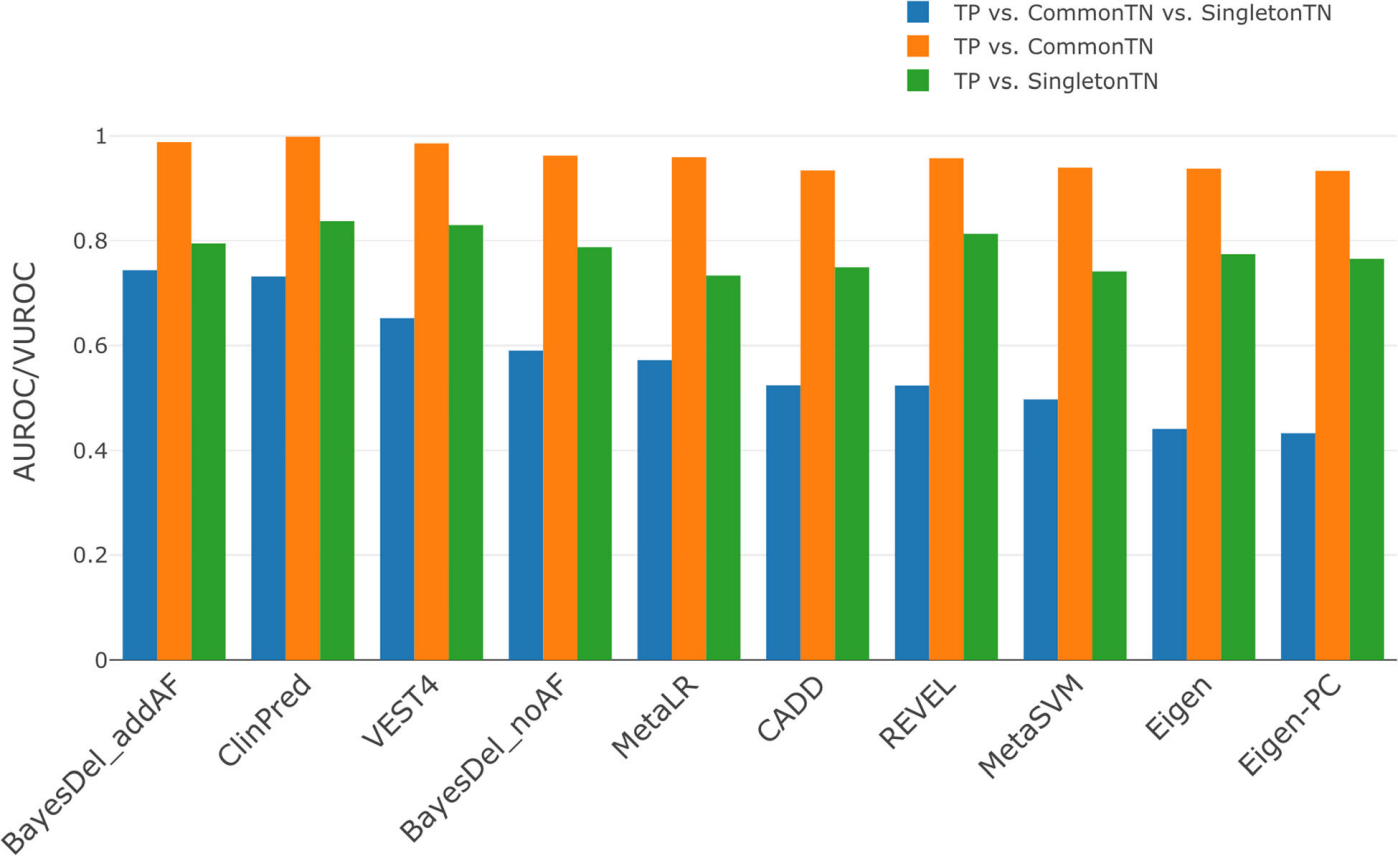
AUROC/VUROC scores for the top 5 deleterious prediction scores

Finally, we compare the performance of the 45 deleteriousness prediction scores and conservation scores. We first collected a test set with true positive (TP) observations obtained from ClinVar between date 20200102 to 20200506 and with true negative (TN) observations obtained from gnomAD v2.1.1 hg38 in genomic locations nearby the TP SNVs (Additional file 4: Supplementary methods). In total, we obtained 3113 missense SNVs as our TP group, and 55,914 missense SNVs as our TN group. Because the selection of TN controls is debatable as to whether to use very rare SNVs or to use common ones [54], we further divided our 55,914 TN SNVs into two groups. The first group (CommonTN; *n* = 1211) contains SNVs with AF in gnomAD greater than 1%. The second group (SingletonTN; *n* = 54,703) contains singleton SNVs in gnomAD. We then calculated the area under the receiver operating characteristic (AUROC) for each score: one using TP vs. CommonTN and the other using TP vs. SingletonTN (Fig. 4, Additional file 2: Table S6). The top five performing scores for TP vs. CommonTN are ClinPred and BayesDel_addAF, VEST4, BayesDel_noAF, and MetaLR, while that for TP vs. SingletonTN are ClinPred, VEST4, REVEL, MutPred, and BayesDel_addAF. Interestingly, except for VEST4 and MutPred, all other scores are ensemble scores. As expected, the best AUROC for SingletonTN as control (0.8374) is substantially lower than it for CommonTN as control (0.999), highlighting the importance of future tools to provide better discriminatory power for rare benign SNVs.

As we expect that the SingletonTN group, in general, has a higher probability of being mildly deleterious than the CommonTN group, a score that can correctly distinguish the functional impact of CommonTN and SingletonTN should be more useful in the context of WES or WGS studies. Here, we extended the two-class AUROC to a 3-class volume under the ROC surface (VUROC) measure, which can simultaneously evaluate TP vs. SingletonTN vs. CommonTN. The resulting VUROC score represents the probability of correctly ranking the three test groups. A complete random guess (noninformative score) will have a VUROC of 0.167. Using a custom Python script, we calculated the VUROC for each of the 45 deleterious scores (Fig. 4, Additional file 2: Table S6). The top five performing scores are BayesDel_addAF (VUROC = 0.7443), ClinPred (VUROC = 0.7322), VEST4 (VUROC = 0.6525), BayesDel_noAF (VUROC = 0.5905), and MetaLR (VUROC = 0.5722). Again, except for VEST4, all other scores are ensemble scores.

## Conclusions

In conclusion, we present dbNSFP v4, a significant improvement over v3 over the past 4 years, as to supporting transcript-specific predictions and annotations, convenience to use (GUI support, joint-query of attached databases, and web-service), and double the number of deleteriousness prediction scores as to nsSNV. dbNSFP will continue to serve the community of medical geneticists as to providing comprehensive and easily-accessible tools for functional annotations and predictions for SNVs that cause amino acid changes and splicing changes.

## Supplementary Information

**Additional file 1: Table S1.** A summary of functional prediction scores and conservation scores in dbNSFP v4.1.

**Additional file 2: Table S2.** Nonmissing counts of ssSNV, nsSNV and 45 scores per chromosome. **Table S3.** Density of rank scores based on 100 bins (bin size = 0.01). **Table S4.** Pearson’s correlation coefficients between rank scores. **Table S5.** Ratio of binary predictions’ agreement between scores. **Table S6.** AUROC/VUROC scores between TP testing set and different TN testing sets for the 45 deleterious prediction scores in dbNSFP v4.1.

**Additional file 3: Fig. S1.** Density plots of rank scores of 45 deleteriousness prediction scores or conservation scores (bin size = 0.01).

**Additional file 4.** Supplementary methods.

## Data Availability

The web service of dbNSFP v4 can be found at http://database.liulab.science/dbNSFP. The downloadable version of dbNSFP v4 can be found at http://database.liulab.science/dbNSFP and https://sites.google.com/site/jpopgen/dbNSFP. All data sources and websites for downloading can be found in Additional file 1: Table S1.

## Abbreviations

dbNSFP: Database for nonsynonymous SNPs’ functional predictions
SNV: Single nucleotide variant
WES: Whole-exome sequencing
WGS: Whole-genome sequencing
VEP: Variant Effect Predictor
nsSNV: Nonsynonymous SNV
ssSNV: Splice site SNV
HGVS: Human Genome Variation Society
WGSA: WGS Annotator
TSL: Transcript support level
GUI: Graphic user interface
AA: Amino acid
UPGMA: Unweighted Pair Group Method with Arithmetic Mean
AUROC: Area under the receiver operating characteristic
VUROC: Volume under the ROC surface
