# Supplementary material for: dbNSFP v4: a comprehensive database of transcript-specific functional predictions and annotations for human nonsynonymous and splice-site SNVs
Source: Genome Med. 2020 Dec 2;12:103. doi: 10.1186/s13073-020-00803-9 (PMC7709417; doi:10.1186/s13073-020-00803-9)
Supplement: Supplementary file 1 — Additional file 1: Table S1. A summary of functional prediction scores and conservation scores in dbNSFP v4.1. [file 13073_2020_803_MOESM1_ESM.docx]

**Table S1: A summary of functional prediction scores and conservation scores in dbNSFP v4.1.**

| **Score** | **Training data** | **Information used** | **Prediction model** | **Website** |
| --- | --- | --- | --- | --- |
| PolyPhen2-HDIV | 5564 Mendelian disease mutations and 7539 divergence SNVs from close mammalian homolog proteins | eight sequence-based and three structure-based predictive features | naive Bayes classifier | http://genetics.bwh.harvard.edu/pph2/ |
| PolyPhen2-HVAR | 22196 disease associated SNVs and 21119 common SNVs | same as above | same as above | same as above |
| SIFT | 1750 deleterious and 2254 tolerant nsSNVs of E. coli LacI gene | sequence homology based on PSI-BLAST | position specific scoring matrix | http://provean.jcvi.org/downloads.php |
| SIFT4G 2.4 | same as above | same as above | same as above | http://sift.bii.a-star.edu.sg/sift4g/ |
| MutationTaster 2 | SNVs from 1000 G (1000 Genomes Project), HGMD | conservation, splice site, mRNA features, protein features; regulatory features | naive Bayes classifier | http://www.mutationtaster.org/ |
| LRT | coding sequences of 32 vertebrate species | sequence homology | likelihood ratio test of codon neutrality | http://www.genetics.wustl.edu/jflab/lrt_query.html |
| MutationAssessor r3 | SNVs from COSMIC database | sequence homology of protein families and sub-families within and between species | combinatorial entropy formalism | http://mutationassessor.org/r3/ |
| FATHMM v2.3 | SNVs from HGMD and UniProt | sequence homology | hidden Markov models | http://fathmm.biocompute.org.uk/ |
| PROVEAN 1.1 | SNVs from UniProt/HUMSAVAR | sequence homology | Delta alignment score | http://provean.jcvi.org/index.php |
| VEST4 | SNVs from HGMD and the Exome Sequencing Project | 86 sequence features | Random Forest | http://karchinlab.org/apps/appVest.html |
| fathmm-MKL coding | SNVs from HGMD and 1000G | conservation, epigenomic signals | multiple kernel learning | http://fathmm.biocompute.org.uk/ fathmmMKL.htm |
| fathmm-XF | Pathogenic SNVs from HGMD and rare neutral SNVs from the 1000 Genomes Project (156,775 coding) | 27 feature groups, 4 conservation feature groups | Forward selection of multiple kernels | http://fathmm.biocompute.org.uk/fathmm-xf/ |
| MetaSVM | 36,192 SNVs from UnPprot | 9 prediction scores and allele frequencies in 1000G | radial kernel support vector machine | https://sites.google.com/site/jpopgen/dbNSFP |
| MetaLR | same as above | same as above | logistic regression | same as above |
| CADD v1.6 | 15 million human-derived fixed or near-fixed SNVs and 15 million matching simulated non-observed SNVs | > 60 diverse annotations | logistic regression | http://cadd.gs.washington.edu/ |
| DANN | 16,627,775 “observed” variants and 49,407,057 “simulated” variants | 63 annotations (949 features) | deep neural network | https://cbcl.ics.uci.edu/public_data/DANN/ |
| fitCons-i6 | genomes of 54 unrelated human individuals | epigenomic signals of GM12878, H1-hESC and HUVEC | INSIGHT (Inference of Natural Selection from Interspersed Genomically coHerent elemenTs) | http://compgen.bscb.cornell.edu/fitCons/ |
| fitCons-gm | same as above | epigenomic signals of GM12878 | same as above | same as above |
| fitCons-h1 | same as above | epigenomic signals of H1-hESC | same as above | same as above |
| fitCons-hu | same as above | epigenomic signals of HUVEC | same as above | same as above |
| LINSIGHT | same as above | 48 genomic features | linear INSIGHT | https://github.com/CshlSiepelLab/LINSIGHT |
| GenoCanyon v1.0.3 | 12,580,197 genomic locations without labels | 22 annotations for conservation | an unsupervised learning model | http://genocanyon.med.yale.edu/index.html |
| Eigen v1.1 | 76.7 million coding non-synonymous variants | 4 deleteriousness prediction scores, 8 conservation scores, allele frequencies from 4 populations | an unsupervised spectral approach | http://www.columbia.edu/~ii2135/eigen.html |
| Eigen-PC v1.1 | same as above | same as above | an unsupervised spectral approach | same as above |
| M-CAP v1.3 | 63,418 pathogenic variants from HGMD and 3,268,665 predominantly benign variants from ExAC | 9 deleteriousness prediction scores, 7 conservation scores, 298 features from 99 genomes | gradient boosting trees | http://bejerano.stanford.edu/MCAP/ |
| REVEL | 6,182 disease SNVs from HGMD and 281,972 putatively neutral SNVs | 18 deleteriousness prediction scores from 13 tools | random forest | https://sites.google.com/site/revelgenomics/ |
| MutPred v1.2 | 39,218 disease SNVs from HGMD and 26,439 putatively neutral SNVs from Swiss-Prot | SIFT and a gain/loss of 14 structural and functional properties | random forest | http://mutpred.mutdb.org/ |
| MVP | 32,074 disease SNVs from HGMD, UniProt and ClinVar, 86,620 neutral SNVs from UniProt, DiscovEHR and CADD training data | Local context, amino acid constraint, conservation scores, protein structure and interaction, gene mutation intolerance, 10 deleteriousness prediction scores | ResNet | https://github.com/ShenLab/missense |
| MPC | 404 pathogenic missense SNVs from ClinVar, 88,083 neutral SNVs with MAF>1% in ExAC | the missense depletion of the region, missense badness, PolyPhen-2, BLOSUM, and Grantham scores | logistic regression | ftp://ftp.broadinstitute.org/pub/ExAC_release/release1/regional_missense_constraint/ |
| PrimateAI v0.2 | 380,000 common missense variants from humans and six non-human primate species | amino acid sequence flanking the variant of interest and the orthologous sequence alignments in other species | deep residual neural network | https://github.com/Illumina/PrimateAI |
| DEOGEN2 | 27 606 deleterious SNVs and 38 285 neutral SNVs from Humsavar16 | PROVEAN, conservation index, protein folding predictions, domain-oriented feature, interaction patches, gene variation intolerance, pathway features | random forest | https://deogen2.mutaframe.com/ |
| ALoFT 1.0 | 397 benign premature stop variants (in 380 genes), 3300 dominant premature stop variants (in 136 genes), and 5342 recessive premature stop mutations (in 796 genes) | 108 features, including domain and structure features, NMD, network features, evolutionary features, allele frequencies, conservation | random forest | http://aloft.gersteinlab.org/ |
| BayesDel v1 | 39,395 pathogenic variants and 39,978 neutral variants from ClinVar and UniProtKB | 9 deleteriousness prediction scores and maximum minor allele frequency across populations | naïve Bayesian model | http://fengbj-laboratory.org/BayesDel/BayesDel.html |
| ClinPred | 7,059 benign and 4,023 pathogenic SNVs from ClinVar | allele frequencies and 16 deleteriousness prediction scores | The higher of the two probability scores (random forest and gradient boosted decision tree) | https://sites.google.com/site/clinpred/home |
| LIST-S2 | 48,142 rare SNVs in ExAC (MAF between 0.015% to 0.03%) assumed to be deleterious, 24,096 common SNVs in ExAC (MAF>=0.5%) assumed to be benign | conservation | Bayes rules | https://precomputed.list-s2.msl.ubc.ca/ |
| SiPhy | genomes of 29 mammals | multiple alignments | inferring nucleotide substitution pattern per site | https://www.broadinstitute.org/mammals-models/29-mammals-project-supplementary-info |
| GERP++ | genomes of 34 mammals | multiple alignments and phylogenetic tree | maximum likelihood evolutionary rate estimation | http://mendel.stanford.edu/SidowLab/downloads/gerp/ |
| phyloP100way_vertebrate | genomes of 100 vertebrates | same as above | distributions of the number of substitutions based on a phylogenetic hidden Markov model | http://hgdownload.soe.ucsc.edu/goldenPath/hg38/phyloP100way/ |
| phyloP30way_mammalian | genomes of 30 mammals | same as above | same as above | http://hgdownload.soe.ucsc.edu/goldenPath/hg38/phyloP30way/ |
| phyloP17way_primate | genomes of 17 primates | same as above | same as above | http://hgdownload.soe.ucsc.edu/goldenPath/hg38/phyloP17way/ |
| phastCons100way_vertebrate | genomes of 100 vertebrates | same as above | two-state phylogenetic hidden Markov model | http://hgdownload.soe.ucsc.edu/goldenPath/hg38/phastCons100way/ |
| phastCons30way_mammalian | genomes of 30 mammals | same as above | same as above | http://hgdownload.soe.ucsc.edu/goldenPath/hg38/phastCons30way/ |
| phastCons17way_primate | genomes of 17 primates | same as above | same as above | http://hgdownload.soe.ucsc.edu/goldenPath/hg38/phastCons17way/ |
| bStatistic | genomes of human, chimp, gorilla, orangutan and macaque | same as above | model of Nordborg et al. and Hudson and Kaplan for estimating expected reduction in nucleotide diversity at a neutral site due to purifying selection | http://www.phrap.org/othersoftware.html |
