# Supplementary material for: dbNSFP v4: a comprehensive database of transcript-specific functional predictions and annotations for human nonsynonymous and splice-site SNVs
Source: Genome Med. 2020 Dec 2;12:103. doi: 10.1186/s13073-020-00803-9 (PMC7709417; doi:10.1186/s13073-020-00803-9)
Supplement: Supplementary file 3 — Additional file 3: Fig. S1. Density plots of rank scores of 45 deleteriousness prediction scores or conservation scores (bin size = 0.01). [file 13073_2020_803_MOESM3_ESM.docx]

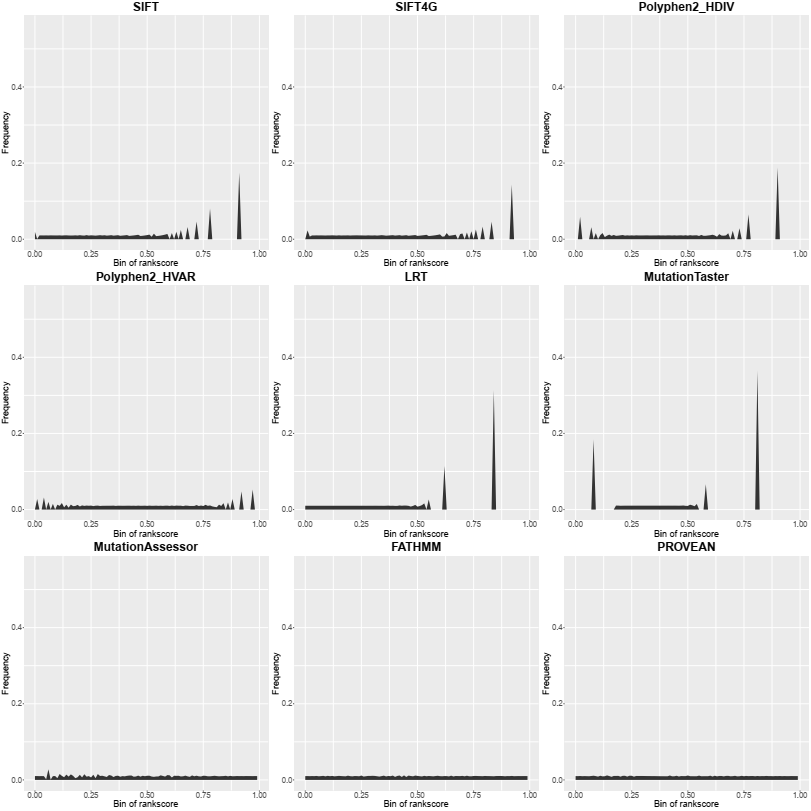

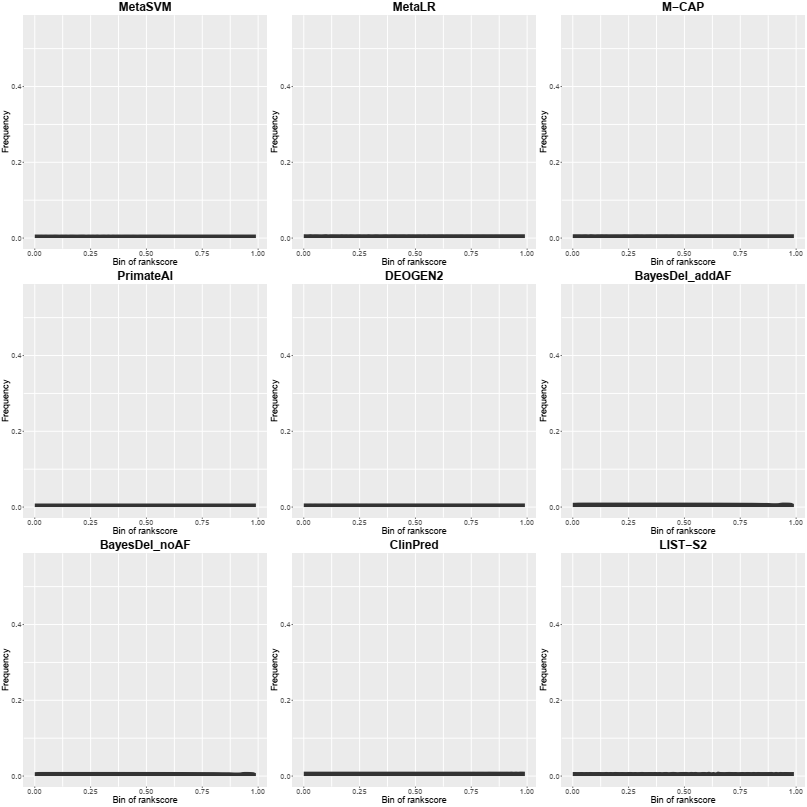

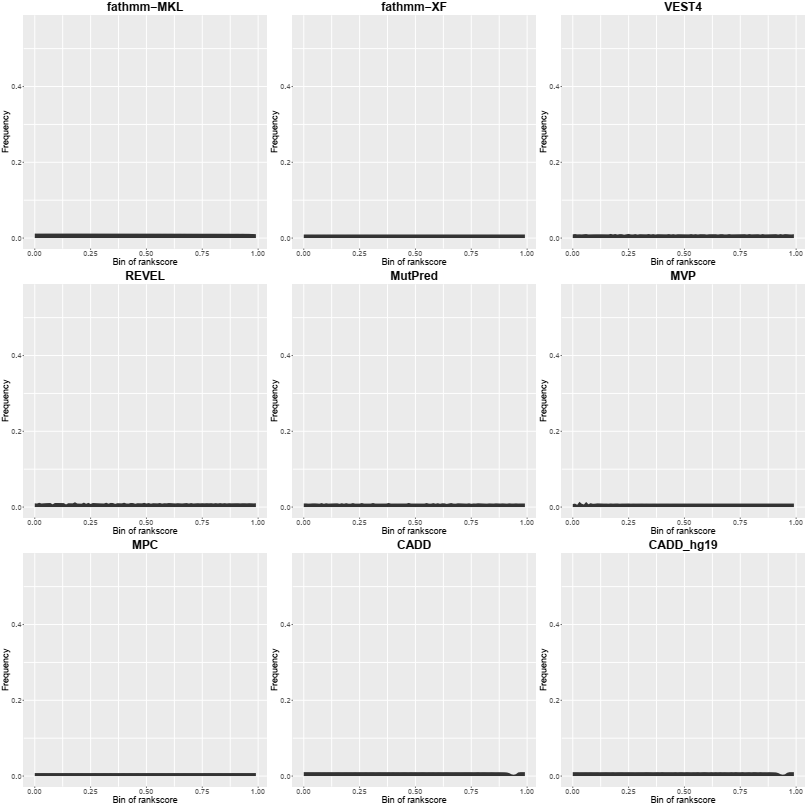

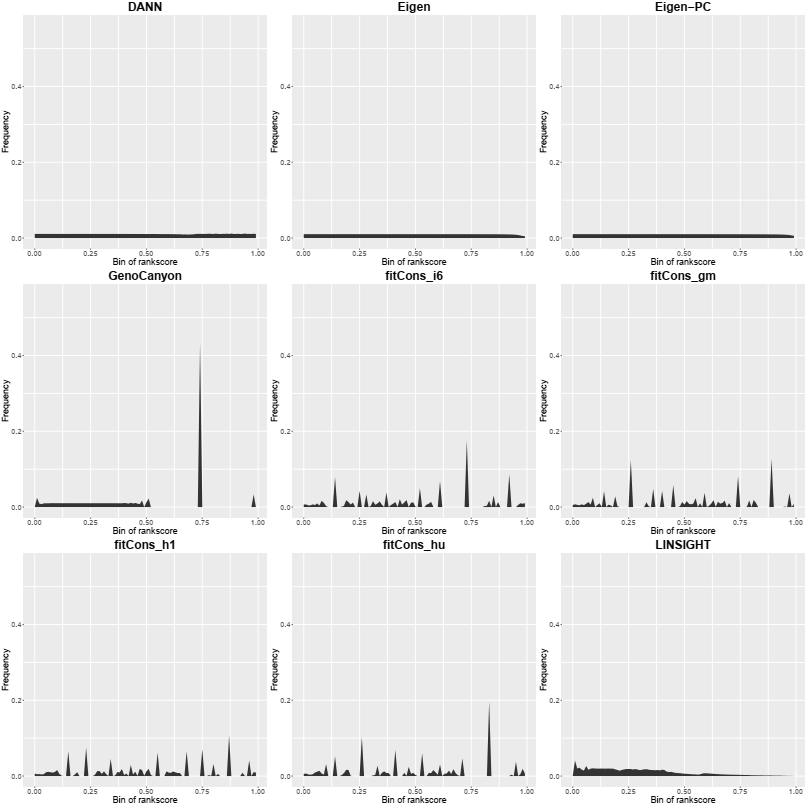

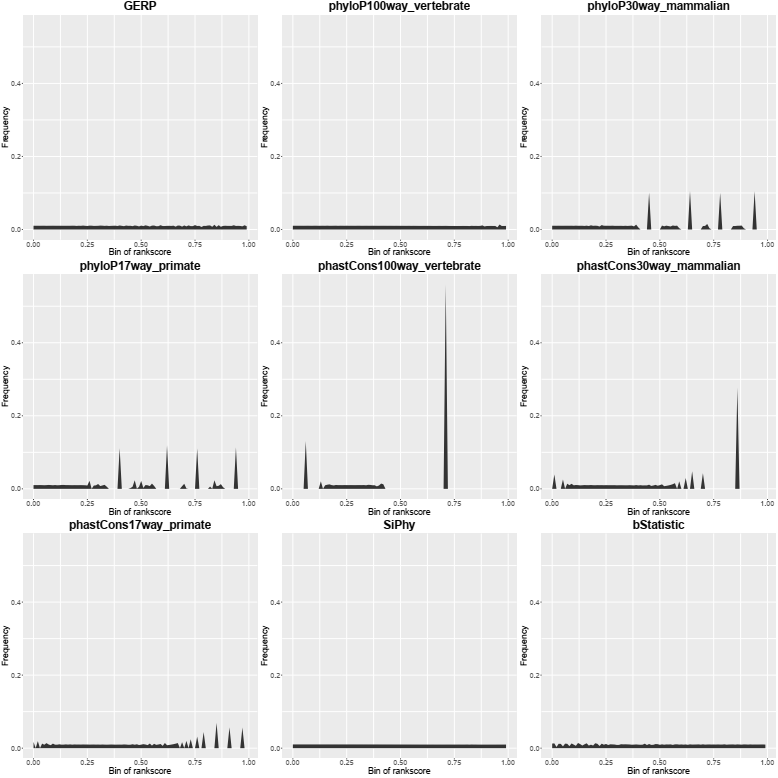


**Fig. S1**: Density plots of rank scores of 45 deleteriousness prediction scores or conservation scores (bin size = 0.01).
