# Supplementary material for: dbNSFP v4: a comprehensive database of transcript-specific functional predictions and annotations for human nonsynonymous and splice-site SNVs
Source: Genome Med. 2020 Dec 2;12:103. doi: 10.1186/s13073-020-00803-9 (PMC7709417; doi:10.1186/s13073-020-00803-9)
Supplement: Supplementary file 4 — Additional file 4. Supplementary methods. [file 13073_2020_803_MOESM4_ESM.docx]

**Supplementary methods**

**Curation of testing data**

We downloaded ClinVar version 20200506 and removed insertion/deletion and all variants in version 20190102. Then, only missense SNVs with at least 1 star were kept. 'Pathogenic' and 'Likely_pathogenic' SNVs were used as true positive (TP) test set (n = 3,113).

We downloaded gnomAD v. 2.1.1 liftover version from <https://gnomad.broadinstitute.org/downloads> and the SNVs in gnomAD were selected if matching any of the two criteria: 1) if allele frequency of the SNV > 1% and within 20kb of any SNVs in TP set 2) if allele count =1 (singleton) and within 1kb of any SNVs in TP set. Using these criteria, we identified 54,940 singleton SNVs from gnomAD which included 237 SNVs that are seen in TP set. After removing these 237 SNVs, there are 54,703 left as of SingletonTN set. Additionally, we identified 1,211 common SNVs (AF > 1%) from gnomAD, which is our CommonTN set.

In each comparison, scores were removed if missing > 0.2 for any of the categories. Four scores were removed based on this criterion: M-CAP (CommonTN 99.5% missing); MutPred (CommonTN 99.5% missing); MVP (CommonTN 99.9% missing); LINSIGHT (All categories > 20% missing).

**AUROC/VUROC analysis**

Area under the ROC curve (AUROC) scores were calculated using function *metrics.auc* from scikit-learn package in Python 3.7.4.

Volume under the ROC surface (VUROC) is a three-dimensional extension of AUROC. It represents the probability of correctly ranking the three testing groups. For the three testing groups TP, SingletonTN and CommonTN, we expect their deleteriousness from low to high is TP, SingletonTN and CommonTN. So we have VUROC:

$$VUROC =\Pr\left\{ CommonTN< SingletonTN< TP \right\}$$

Based on the definition, we used a custom Python script to calculate VUROC. First, we randomly selected 1,000,000 samples from each of the three testing groups with replacement to construct 1 million lists with length 3. Then, the VUROC can be calculated by formula:

$$VUROC = \frac{N_{correct}+ 0.5\times N_{1=}+ 0.167\times N_{2=}}{N_{BS}}$$

where $N_{correct}$ is the number of list with correct order. To account for equal predictions between neighboring scores, we added a weight 0.5 to lists with one equal pair ($N_{1=}$) and added a weight 0.167 to lists with 2 equal pairs ($N_{2=}$). This weight sum is then divided by the number of total bootstrap resampling ($N_{BS}$).
